# The Double-Edged Sword of Total Antioxidant Capacity: Clinical Significance and Personal Experience

**DOI:** 10.3390/antiox13080933

**Published:** 2024-08-01

**Authors:** Andrea Silvestrini, Antonio Mancini

**Affiliations:** 1 Dipartimento di Scienze Biotecnologiche di Base, Cliniche Intensivologiche e Perioperatorie, Università Cattolica del Sacro Cuore, 00168 Rome, Italy; 2 Dipartimento di Medicina e Chirurgia Traslazionale, Università Cattolica del Sacro Cuore, Largo Francesco Vito, 1, 00168 Rome, Italy

**Keywords:** distress, metabolic syndrome, infertility, oxidative stress, total antioxidant capacity

## Abstract

Oxidative stress (OS) could be a condition underlying several human diseases, despite the physiological role of reactive oxygen species (oxidative eustress). Therefore, antioxidant compounds could represent a modulatory mechanism for maintaining a proper redox balance and redox signaling. When antioxidants are insufficient or overwhelmed, OS ensues, causing multiple damages at molecular, tissue, and cellular levels. This study focuses on the role of total antioxidant capacity (TAC) as a biomarker to be interpreted according to several clinical scenarios. After a brief description of various assay methods to elucidate terminology and physiopathological roles, we focus on the hormonal influence on TAC in blood plasma and other biological fluids, as different endocrine systems can modulate the antioxidant response. Furthermore, OS characterizes several endocrinopathies through different mechanisms: an inadequate antioxidant response to an increase in reducing equivalents (reductive distress) or a marked consumption of antioxidants (oxidative distress), which leads to low TAC values. An increased TAC could instead represent an adaptive mechanism, suggesting a situation of OS. Hence, the clinical context is fundamental for a correct interpretation of TAC. This review aims to provide the reader with a general overview of oxidative stress in several clinical examples of endocrine relevance, such as metabolic syndrome, non-thyroid illness syndrome, hypopituitarism, and infertility. Finally, the impact of dietary and surgical interventions on TAC in the model of metabolic syndrome is highlighted, along with personal experience.

## 1. Introduction: Redox Homeostasis and Oxidative Stress Biomarkers

**Figure 1:**
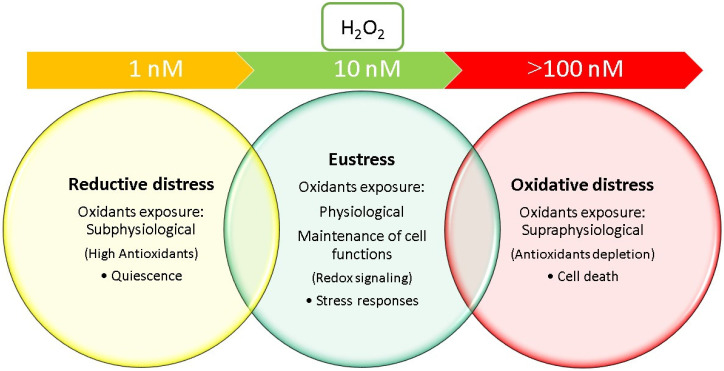
Redox eustress (green) and transition to oxidative (red) and reductive (yellow) distress. The levels of oxidant exposure (e.g., H_2_O_2_) range from physiological (i.e., 10 nM) (redox eustress) at a minimum (H_2_O_2_ less than 1 nM) to very high levels (H_2_O_2_ more than 100 nM). Accordingly, the hormetic range includes transitional phases of exposure from physiological (green) to “subphysiological” (yellow) or “supraphysiological” (red), which can lead to reductive distress or oxidative distress, respectively.

Oxidative stress (OS) may occur when there is an imbalance between the production of reactive oxygen species (ROS) and the cellular ability to counteract them with antioxidants. ROS are highly reactive molecules containing oxygen that can cause damage to proteins, lipids, and DNA if their levels overwhelm antioxidant defense. Data concerning antioxidants supplementation trials and markers of OS are consistent with a new definition of OS as the “disruption of redox signaling and control” [1]. Hydrogen peroxide (H_2_O_2_) and superoxide anion radical are key components acting as redox signaling agents. Several cellular reactions naturally produce oxidants as by-products of normal metabolism and immune system function; excessive oxidants production or inadequate antioxidant defenses can lead to oxidative distress. Interestingly, H_2_O_2_, when confined to a physiological concentration (i.e., 10 nM), is utilized as a second messenger in redox biology [2] and in this context can be defined as “oxidative eustress” (Figure 1). Higher H_2_O_2_ concentrations (i.e., more than 100 nM) drives different cellular outcomes that can lead to degeneration called oxidative distress that can lead to cell death [3]. According to the “redox hypothesis”, oxidative stress arises from the disruption of thiol redox circuits, which play a role in cell signaling and physiological regulation [4]. However, OS can be seen as a double edged sword: when supraphysiological concentration of oxidants are produced, it is defined as “oxidative distress”; when subphysiological exposure of oxidants are present, it is defined as “reductive distress” (Figure 1). Reductive distress is a condition marked by the excessive accumulation of reducing equivalents (e.g., GSH, NADH), which can interfere with several biochemical pathways necessary for cell proliferation. The clinical implications of oxidative damage (oxidative distress) include its involvement in various diseases and conditions [5]. Accordingly, oxidative distress is implicated in the development and progression of neurodegenerative disorders such as Alzheimer’s disease, Parkinson’s disease [6], and amyotrophic lateral sclerosis. The accumulation of ROS can damage neurons and contribute to the deterioration of cognitive and motor functions [1,3].

Oxidative distress plays a role in the development of cardiovascular diseases, including atherosclerosis, hypertension, and heart failure. Accordingly, oxidants can damage blood vessels, promote inflammation, and contribute to the formation of plaque in the arteries. Oxidative distress is associated with the initiation, promotion, and progression of cancer [7]. The ROS can damage DNA, leading to mutations that promote the development of cancerous cells. Additionally, oxidants can contribute to tumor growth and metastasis by promoting angiogenesis and inhibiting apoptosis [7]. Moreover, oxidative distress can exacerbate inflammation by activating signaling pathways that promote the release of pro-inflammatory cytokines and chemokines. Chronic inflammation associated with conditions such as rheumatoid arthritis, inflammatory bowel disease, and asthma is linked to increased oxidative distress. Moreover, OS is believed to contribute to the aging process by causing cumulative insults and damage to cells and tissues over time. The decline in antioxidant defenses and the accumulation of oxidative damage are thought to play a role in age-related decline in physiological function and the development of age-related diseases (e.g., cardiovascular diseases, neurodegenerative diseases, chronic obstructive pulmonary disease, and chronic kidney disease) [8]. Furthermore, oxidative distress is involved in the pathogenesis of diabetes and its complications. High levels of glucose can induce the production of oxidants, leading to oxidative damage to pancreatic beta cells, insulin resistance, and damage to blood vessels and organs. 

Despite this ubiquitous physiopathological mechanism, some aspects still require further investigation, especially from a diagnostic and therapeutic point of view, since many pharmacological/nutraceutical approaches remain empirical. Therefore, a more rational approach should be based on factors that modulate antioxidant systems in vivo.

Overall, oxidative distress is a crucial aspect in the development and progression of numerous diseases and conditions, making it an important target for therapeutic interventions aimed at reducing oxidative damage, restoring antioxidant balance and redox signaling for the maintenance of cell functions. Strategies to mitigate oxidative distress include lifestyle modifications (such as a healthy diet with antioxidants enrichments, regular exercise, and avoidance of smoking and alcohol consumption) and pharmacological interventions targeting oxidative stress pathways. Detecting oxidative stress typically involves assessing the levels of reactive oxygen species (ROS) or the extent of oxidative damage to biomolecules such as lipids, proteins, and DNA. 

Below are some commonly used methods for detecting oxidative stress, while we refer to our recent review for a more detailed description of various assays [9].

Antioxidant enzymes such as superoxide dismutase (SOD), catalase, and glutathione peroxidase play a crucial role in neutralizing oxidants. Among protein transcription factors, Nrf2 (NF-E2-related factor 2) acts as a master regulator of cellular responses against environmental stresses [10]. Oxidative distress triggers the activation of various kinases (e.g., MAPK, ERK, p38). These kinases phosphorylate both Keap1 (Kelch-like ECH-associated protein) and Nrf2, disrupting the Keap1-Nrf2 complex and promoting the translocation of Nrf2 to the nucleus. In the nucleus, Nrf2 forms a complex with Maf proteins. The Nrf2/Maf heterodimers then bind directly to antioxidant response elements (AREs) within the promoters of Nrf2 target genes, coordinating the expression of antioxidant gene products. Thus, the induction of antioxidant enzymes through the antioxidant response element (ARE) is mediated by Nrf2. Accordingly, when oxidative stress conditions occur, specific stress-sensing cysteine residues in Keap1 are modified, causing a conformational change. This change leads to the stabilization, accumulation, and nuclear translocation of Nrf2, which regulates ARE-containing cytoprotective genes and transactivates a battery of antioxidant enzymes [11]. Therefore, the measurement of the activity of these enzymes can provide indirect information about oxidative stress levels. Enzyme activity assays typically involve spectrophotometric or colorimetric methods.

Lipid peroxidation is a common consequence of oxidative stress. Biomarkers of lipid peroxidation include malondialdehyde (MDA) and 4-hydroxynonenal (4-HNE), which can be measured using colorimetric or chromatographic methods.

Protein carbonylation and formation of advanced glycation end products (AGEs) are biomarkers of protein oxidation. Techniques such as enzyme-linked immunosorbent assay (ELISA) or Western blotting can be used to quantify protein oxidation.

Oxidative damage to DNA, such as the formation of 8-hydroxy-2′-deoxyguanosine (8-OHdG) as biomarker, can be detected using ELISA or immunohistochemistry methods.

Glutathione (GSH) is a critical antioxidant in the body, playing a key role in protecting cells from oxidative stress by neutralizing oxidants. When GSH concentration is low in tissues, the release of GSH into the plasma also decreases. Accordingly, when oxidized glutathione (GSSG) levels increase in cells, it also increases in the plasma. Thus, the balance of plasmatic GSH and GSSG can be a useful indicator of oxidative stress. Indeed, this balance reflects both the ability of GSH to protect against oxidative reactions and the generation of GSSG from those reactions [1]. Interestingly, plasmatic GSH redox becomes progressively oxidized with age after 45 years [1]. Adequate levels of GSH are associated with reduced oxidative damage and a lower risk of age-related diseases.

One of the most widely used assay to reveal OS in plasma is total antioxidant capacity (TAC). This assay can provide valuable information about systemic oxidative stress levels. Accordingly, TAC assays measure the ability of biological samples to neutralize ROS. Common TAC assays include the ferric reducing antioxidant power (FRAP) assay, oxygen radical absorbance capacity (ORAC) assay, and Trolox equivalent antioxidant capacity (TEAC) assay [9]. The presence of endogenous chain-breaking antioxidants (e.g., GSH, lipoic acid, bilirubin, coenzyme Q10, uric acid) induces a lag time (the “lag phase”) in the generation of radical species, whose duration is proportional to the concentration of antioxidants. Thus, the total antioxidant capacity afforded by chain-breaking antioxidants is often expressed as the length of lag phase (LAG) reported in seconds.

Various oxidative stress biomarkers can be measured in blood or urine samples, including ROS levels, antioxidant enzyme activity, and markers of oxidative damage to biomolecules [12]. It is important to consider that each method has its advantages and limitations, and the choice of detection method may depend on factors such as the specific biomolecule being assessed, the sample type, and the experimental objectives [9]. Additionally, interpreting oxidative stress measurements requires careful consideration of potential confounding factors and the biological context of the samples being analyzed. Moreover, some parameters are also influenced by diurnal variation and fasting or feeding status as reported in plasma for GSH/GSSG and cysteine/cystine [13]; therefore, a standardization of sample collection to avoid other confounding factors is recommended. 

Among the influencing factors that modulate the response to OS, hormonal secretions also play a key role if already underestimated. Therefore, we reviewed the main endocrinological conditions associated with OS and TAC as well as the usefulness of assays in several clinical contexts. Thus, we mainly focused on evaluating this index in hormonal and metabolic diseases, providing several clinical examples of endocrine relevance where the role of oxidative stress is well recognized. As discussed above, low TAC can reflect an inadequate response, or vice versa, an augmented consumption of antioxidants. Conversely, high values of TAC may suggest an efficient defense system or reveal an oxidative condition. This article discusses the double-edged sword of TAC values and their interpretation in various clinical contexts.

## 2. Hormonal Modulation

Oxidative stress (OS), as defined above, is an important pathogenetic mechanism in several endocrine/metabolic diseases. Moreover, inflammation and OS are closely related processes, exemplified in obesity conditions and in cardiovascular diseases [14]. OS is also related to hormonal derangement in a reciprocal mode. Despite the abundant literature in this field, many aspects of hormones’ role in regulating antioxidant synthesis and activity remain unclear. We have already reviewed experimental data, in vivo and in vitro, on the effects of the different pituitary-dependent hormonal axes on antioxidant levels, trying to provide a comprehensive view of hormones that also have antioxidant properties, including the lipophilic antioxidant Coenzyme Q10 (CoQ10), strictly related to thyroid function and total antioxidant capacity [15].

Among the various hormonal effects that can influence the antioxidant balance, thyroid hormones (free thyroxine, fT4; free tri-iodothyronine, fT3) play particularly important roles, since both hyperthyroidism and hypothyroidism have been shown to be associated with OS in animals and humans [16]. Thyroid hormones per se can act as oxidants and produce DNA damage, due to their phenolic ring similar to that of estrogens [17]. Many other mechanisms have been described elsewhere [18]; however, the two thyroid hormones (i.e., fT4 and fT3) may exert differential effects, with a specificity in tissue response, mainly regarding enzymatic antioxidants. As non-enzymatic antioxidants are involved, an inverse correlation between fT4 and CoQ10 (lipophilic antioxidant also endowed with energetic properties as component of the mitochondrial respiratory chain) is already reported [19]; TAC, on the contrary, has been reported to be lower in hypothyroid patients [15]. The physiological production of radicals is related to steroidogenesis [20], but OS has been demonstrated in adrenal-ectomized rats [21] and as a component of the “stress response” as an adaptive mechanism to stressful conditions [22]. In addition, testosterone has been suggested to exert pro-oxidant effects in experimental models [23], even if a protective effect has been shown in other models, such as neuronal cells [24]. Thus, the effects of estrogens appear to be more complex [25,26,27]. 

Taken together, despite some inconsistencies, these data underline the importance of oxidative stress in several pituitary-dependent disorders, suggesting the possible clinical usefulness of TAC determination as an index of OS. In humans, indirect data about hormonal influence on antioxidants can be obtained by evaluating their levels in models of endocrine hypo-secretion. For instance, hypogonadism is associated with an increased risk for cardiovascular disease, possibly linked to OS. To investigate the role of gonadal steroids in systemic antioxidant regulation, our research group has determined plasma CoQ10 concentrations and TAC in postsurgical hypopituitaric subjects. The CoQ10 levels were measured by high-performance liquid chromatography and TAC by spectroscopy using the ABTS system as described previously [28]. CoQ10 levels were significantly lower in isolated hypogonadism than in normogonadism. Testosterone treatment, performed in those patients with isolated hypogonadism, induced a significant enhancement both in CoQ10 level and LAG values. In fact, CoQ10 and LAG values correlated significantly, suggesting an interrelationship between different antioxidants. Thus, these data suggest that hypogonadism could represent a condition of oxidative stress, in turn related to augmented cardiovascular risk [29]. Other studies were in agreement with our observations [30,31]. In the last paper, however, despite lower TAC than controls, the picture of oxidative markers was not univocal; myeloperoxidase did not differ from control subjects; furthermore, replacement therapy did not influence total lipid hydroperoxide and protein carbonyl compounds; therefore, the authors suggested that hypogonadism do not represent a situation of “all or none” oxidative stress. Other factors, which further complicate the relation between OS and hypogonadism, can be represented by age and chronic diseases related to low testosterone (T) levels [32] and environmental factors inducing OS and hypogonadism [33]. It must be remembered that OS itself may reduce T synthesis, despite the physiological ROS production that ensues during steroidogenesis [32], but excessive ROS production suppresses transcriptional factors regulating the expression of steroidogenetic enzymes [34]. Reduced T synthesis is also observed in Nrf2 knockout mice with a consequent reduced antioxidant enzymes response to OS [35]. On the other side, a biphasic T effect has been hypothesized: in agreement with our results, TM3 Leydig cells exposed to low-dose T concentrations exhibited increased steroidogenic acute regulatory protein (StAR) expression and reduced ROS and lipid peroxidation [36]. A specular effect (downregulation of StAR, increased lipid peroxidation, and advanced glycation end products) was reported in healthy adult male rats treated with higher T doses [37].

A study, similar to that with hypogonadal patients, was also performed on adrenal disease. In this model, CoQ10 and total antioxidant capacity (TAC) were determined in pituitary-dependent adrenal diseases: ACTH-dependent adrenal hyperplasia (AH), secondary isolated hypoadrenalism (IH) and hypoadrenalism-associated hypothyroidism (multiple pituitary deficiencies, MPH). The CoQ10 levels were significantly lower in IH compared to AH and MPH, with a similar trend when adjusted for cholesterol. In addition, TAC was lower in IH than in AH and MPH, suggesting that adrenal hormones can influence antioxidants. However, since thyroid hormones modulate CoQ10 levels and metabolism, when thyroid deficiency coexists, it seems to have a prevalent influence [38].

Finally, interesting data were obtained in the growth hormone deficiency (GHD) condition. Adult GHD is a disease associated with increased cardiovascular risk (CV) and insulin resistance (IR). Despite being considered a rare disease, it is actually underdiagnosed, since it is investigated only in patients with known hypothalamic-pituitary disease (such as surgery, irradiation, or trauma). OS could be a mechanism underlying both CV and IR risks. In order to investigate plasma antioxidant defenses in such conditions, our research group has studied adults with GHD, compared with controls and metabolic syndrome patients (MetS), evaluating the TAC and CoQ10 levels (in its oxidized and reduced forms) and correlating these data with metabolic and hormonal patterns [39]. In this case–control study, despite the HOMA index being higher in both GHD and MetS, lower LAG levels was only observed in MetS, suggesting an increased consumption of antioxidants in metabolic syndrome patients. Moreover, LAG significantly correlated with uric acid only in MetS, suggesting a different pattern of antioxidants. In conclusion, these data indicate that GHD, although sharing with MetS various metabolic features, including increased HOMA levels, showed a differential pattern of plasma antioxidants, suggesting inadequate reactivity toward radical production rather than antioxidants consumption as evidenced in MetS [39].

In biomedical science, hormesis can be defined as an adaptive biphasic response of cells and the entire organism to moderate stress [40]. In fact, hormesis is a dose–response relationship where low-dose exposures are beneficial, while high doses of the same agent are detrimental. This biphasic/dual dose response, among hundreds of hormesis studies, shows that exposure to low levels confers protection against a more challenging high-level event that confers toxicity. Moreover, it can be claimed that hormesis may produce small amounts of ROS production that could be a crucial part of the adaptive response [41]. A paradigmatic example of this dual response can be evidenced in infertility both in males and females [42]. Despite ROS covering physiological roles, it has been reported that 30–80% of infertility is due to increased ROS or low seminal plasma TAC [43]. More recently, it has been proposed to evaluate the balance between these two factors, studying the oxidation-reduction potential [44]; again, normozoospermic patients have better parameters in pathological semen. Remarkably, the modulation of antioxidants by hormones can also be exerted in seminal plasma. In infertile patients, we observed via a correlation analysis, an inverse correlation between PRL and sperm motility, and a direct correlation of TAC with PRL and fT4, but not with gonadotropins or gonadal steroids. Taken together, these data suggest that systemic hormones may play a role in regulating the seminal antioxidant capacity. This could also be interesting because some hormones, such as thyroid and pituitary hormones, are not usually tested in the initial evaluation of male patients with fertility problems [45]. Interestingly, the role of antioxidant is also determinant in females; despite ROS being a key trigger for ovulation, oocytes need to be protected against ROS, mainly by surrounding cumulus cells [46] and the composition of follicular fluid. In addition, the fluids in the fallopian tubes and the uterus are needed to counteract ROS effects [47]. Among the non-proteic non-enzymatic molecules, a major role has been attributed to glutathione. However, few data are available on TAC measures due to the complex compositions of these fluids [48]. A study compared females defined as “poor responders” to treatments for in vitro fertilization with controls; it showed lower TAC and SOD levels, together with increased follicular fluid MDA. Thus, high levels of OS in poor ovarian responders can be alleviated by GH pretreatment [49]. Interestingly, treatment with growth hormone increased TAC follicular fluid, suggesting a protective role of GH against oxidative stress, as will be extensively described below. 

Despite all these factors and the large wealth of literature about the role of OS in infertility, no clear evidence has been reported for antioxidant treatments, neither for male factors [50] nor for infertile couples [51].

## 3. Low TAC as an Inadequate Antioxidant Response to Oxidative Stress

Remarkably, there is evidence in the literature of age-dependent modulation of antioxidants [1]. Despite the lack of specific studies on the physiological role of antioxidants in healthy humans, many papers discuss the beneficial effects of ROS as second messengers, sensors, modulators of redox metabolism, anti-infective agents, and ultimately, in determining cell fate: life, proliferation, or death/apoptosis [52]. The “redox-homeostasis” is a concept indicating the redox intracellular environment, related to the production and removal of oxidants and is linked to proper cell metabolism and function [53]. On the other hand, the modulation of antioxidants has been deepened in relation to many diseases and, in particular, lifespan periods: the extremes of human life (childhood and aging) are especially interesting under this profile, since they are characterized by inadequate antioxidant response to increased radical production [7]. The extremes of lifespan, i.e., youth and old age, are especially interesting under this profile. The question about childhood is crucial, since obese children are subject to the same chronic oxidative and inflammatory stress, responsible for the onset of all the complications typical of adult age, such as insulin resistance, type 2 diabetes, dyslipidemia, and cardiovascular disease. Nevertheless, in the literature, contrasting results have been reported, even if most studies suggest low antioxidant status in obese children. 

### 3.1. Low TAC in Childhood 

Vehapoglu [54] and co-workers investigated a group of obese pre-pubertal children, in comparison with underweight and normal weight controls. They found elevated inflammatory indexes and decreased antioxidant status in the obese group vs. the other groups [54]. In particular, while metabolic parameters, as expected, were higher in the obese group (fasting glucose, triglycerides, total cholesterol, insulin, HOMA index, C reactive protein, neutrophils, and neutrophil/lymphocyte ratio), lower antioxidants and thiols were observed, with a negative correlation with age and, in the overall population, with the metabolic parameters reported above. Lower carotenoid levels in serum were reported in obese U.S. adolescents with metabolic syndrome, in comparison with a BMI-matched group without metabolic syndrome [55]. Similarly, total plasma antioxidants and α-tocopherol levels were reported in obese children with MetS [56]. Moreover, another study reported alterations in metabolic syndrome, even in a partial form, showing oxidative stress and elevated platelet activation; in this study, low values of vitamin E and TAC were evidenced, while plasma 15-F(2t)-isoprostane levels and protein carbonyls were higher [57]. It has been shown that low levels of Zinc, Vitamins A and E were associated with insulin resistance and inflammation in overweight and obese children [58].

In agreement with these results, in our previous paper, we investigated the relationship between oxidative stress, body composition and metabolic pattern in childhood obesity in comparison with adult obese patients. LAG values in children significantly correlate with % fat mass, waist circumference, and waist/hip ratio. However, mean LAG values were significantly lower than in obese adults [59]. This agrees with a previous finding, where increased activity of enzymatic antioxidants in obese vs. control children were described [60]. Brown et al. [61] did not find significant differences in total antioxidant status and reduced glutathione when comparing normal-weight, overweight and obese adults. Similarly, increased antioxidant capacity was described in obese adults in a Mexican cohort [62]. The apparent discrepancy could be related to the different parameters studied and the duration of oxidative stress. In other words, we can say that children are more susceptible to oxidative stress than adults, possibly due to the incomplete development of the antioxidant system. An inadequate alimentary intake of antioxidants could also be an important factor to consider, since lower contribution of fruit and vegetables is usually present in obese children [63,64]. This is also the rationale for interventional studies, which showed an amelioration of antioxidant status with the appropriate supplementary diet [65,66].

Finally, it has been reported that even in the pre-pubertal period, age-related changes in antioxidant status could be related to metabolic variations observed during very early periods of life when active growth and development require rapid cellular turnover. Therefore, it is not in contrast with the data reported above the elevated level of d-ROMs (reactive oxygen metabolites), urinary 8-hydroxy-2-deoxyguanosine (8-OHdG), and BAP (biological antioxidant potential), which has been described in a group of 2–6 years of age, while they show a trend in decreasing with the progression of age [67]. 

### 3.2. Low TAC in Aging

Interestingly, it is widely accepted that the generation of endogenous oxygen radicals induces cumulative damage, as stated in the original “free radical theory” proposed in 1956 [68]. The progressive damage to nuclear DNA and mitochondrial DNA with increasing age is clearly supported by in vitro and in vivo experimental data [69,70]. Consequently, the ability to respond to this phenomenon is crucial as an anti-aging mechanism. Recent data obtained in *C. elegans* and *Drosophila* confirm this concept, as reported by Finkel and Holbrook [71]. Anyhow, extrapolating these sophisticated experiments to humans is not simple, but some clinical models can help interpret the evaluation of antioxidant capacity in aging individuals. Cardio-tolerance to OS progressively decreases due to the lack of adaptation in antioxidants, particularly antioxidant enzymes, leading to the development of cardiometabolic disorders [1,72,73]. 

Beyond metabolic and cardiovascular diseases, osteoporosis is a major problem during the geriatric period. In this condition, there is a convincing basis that suggests oxidative stress as a pathogenetic factor. ROS production could be responsible for a pH decrease during osteoclastic hyperactivity with a consequent sensitization in sensory nerve fibers, causing back pain [74]. Furthermore, ROS greatly influence the generation and survival of osteoblasts, osteoclasts and osteocytes. Sexual hormones represent an important defense against OS in bone metabolism [75].

With the purpose of evaluating the role of oxidative stress (OS) in male idiopathic osteoporosis, our group has evaluated plasma TAC in patients classified according to age (<65 or ≥65 years), with normal hormone values and in age-matched healthy control subjects [68]. We found slightly increased TAC values in middle-aged patients, compared with age-matched controls, probably expression of a compensatory mechanism to OS; on the contrary, older patients showed significantly lower TAC values in comparison with age-matched controls, suggesting a defective compensatory mechanism and, therefore, an augmented risk for oxidative damage. OS could be a possible mechanism underlying male osteoporosis, both in middle-aged and aged patients, but compensatory mechanisms seem to be defective in the last group [76]. The reduced levels of testosterone and growth hormone can worsen the problem by increasing oxidative stress. Specially acquired growth hormone deficiency can be associated with a higher prevalence of spine fractures [77]. Of particular importance is the concept of hormesis (as mentioned above); it implies that low levels of stress represent the optimal system for activating defense mechanisms [78]. However, the multifactorial decrease in antioxidant response remains an undisputed involved factor.

## 4. Low TAC as an Expression of Antioxidants Consumption

**Figure 2:**
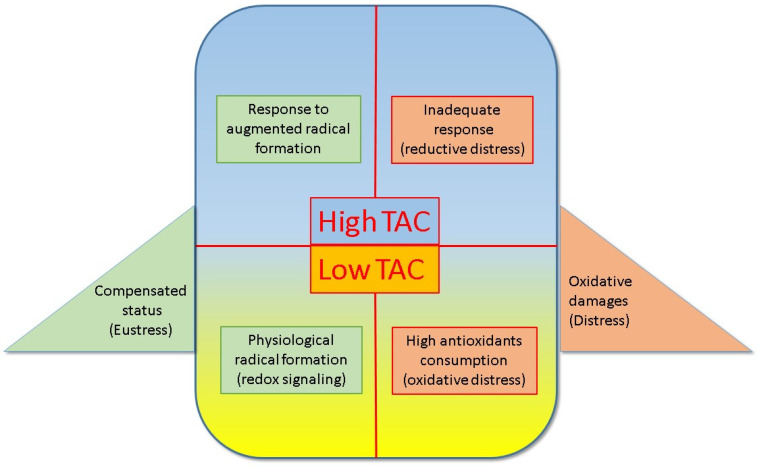
The double-edged sword of total antioxidant capacity (TAC) in several clinical situations. The upper panels show a condition of high TAC, which can be related to augmented antioxidant production (or an adequate response), to increased radical formation (on the left), or an unbalanced antioxidant response that is not able to counteract marked radical production (inadequate response “reductive distress”) on the right. The lower panels depict conditions of low TAC, which could express a physiological status related to low radical production (left side) or, on the contrary, a very high consumption of antioxidants induced by high radical production (on the right), which leads to high antioxidant consumption. Therefore, the panels on the left side (upper and lower) suggest a compensatory status and maintenance of redox signaling the “eustress” condition, while those on the right side (upper and lower) indicate an actual condition of “distress”, which may be harmful to the body at the molecular and tissue levels.

Low plasma TAC levels may not be indicative of an inadequate response, but rather result from antioxidant consumption due to “oxidative distress” (Figure 2). This section seems to include several clinical conditions of oxidative distress, such as low T3 syndrome, chronic obstructive pulmonary disease, and metabolic syndrome.

### 4.1. Low T3 Syndrome 

Low T3 syndrome is the most common picture of the “Non-thyroidal illness syndrome” (NTIS), which represents a response to different acute and chronic diseases in the absence of organic thyroid involvement [79]. It typically manifests in the reduced conversion of thyroxine (fT4) to triiodothyronine (fT3) and is a chronic systemic condition that is still a debated topic. The thyroid adaptation suggests not to treat the condition with replacement therapy; on the contrary, it seems to be a “maladaptation” with negative consequences at the tissue level, as suggested by other reports [80]. The roles of deiodinases, the enzymes responsible for the conversion of T4 to T3, are key points in this context. The presence of OS indexes in NTIS supports the hypothesis that it represents a condition of hypothyroidism at the tissue level and not only an adaptive mechanism to diseases [81].

### 4.2. Chronic Obstructive Pulmonary Disease (COPD) 

Among the chronic diseases with a low-T3 syndrome, there is chronic obstructive pulmonary disease (COPD). It is a complex condition that cannot be considered a lung-related disorder, but rather a systemic disease that is also associated with increased oxidative stress [82]; the aging process complicates the antioxidant status. Few inconclusive data are published about the crosstalk between thyroid and lung function in this condition; therefore, we evaluated the CoQ10 and TAC in COPD patients to reveal the presence of a low-T3 syndrome in COPD and investigated the correlation between thyroid hormones, lung function parameters, and antioxidants [83]. We found significantly lower LAG values, fT3 and fT4 levels, and significantly higher TSH in the whole group of COPD patients versus controls. LAG values significantly correlated with fT3 concentration. Moreover, we found lower LAG and higher CoQ10/cholesterol ratio in the subgroup of patients who presented low-T3 syndrome. These data seem to indicate an increased oxidative stress in low fT3-COPD and the role of fT3 in modulating antioxidant systems. However, low fT3 levels are associated with metabolic indexes of true hypothyroidism, suggesting that elevated CoQ10 expresses a reduced tissue utilization. These data have a clinical relevance, since they might suggest the need for thyroid replacement therapy in such a condition [83].

### 4.3. Metabolic Syndrome (MetS) 

Several studies have been published on the role of oxidative stress (OS) in the development of metabolic syndrome (MetS), especially concerning its cardiovascular complications. Much evidence supports this physiopathological mechanism, which is also linked to carcinogenesis [84,85,86]. In the context of a genetic predisposition to insulin resistance (IR), induced by excessive caloric intake or sedentary lifestyle, it has been hypothesized that diet-induced OS is a factor [87]. The chronic free fatty acids overflow and can worsen insulin sensitivity (the so called “lipotoxicity”), inducing a reduction in insulin-dependent glucose uptake [88]. The insulin receptor substrate phosphorylation is involved in OS-induced IR [89]. Long-term, large-scale, population-based cohort studies have shown that a lower risk of cardiovascular disease is associated with increased levels of various non-enzymatic antioxidants [90], including albumin [91,92], bilirubin [93], glutathione [94], tocopherols [95], Vitamin C [96,97], and carotenoids [98]. As antioxidants represent a network exerting a protective effect in response to radical production, no single molecule can be representative of antioxidant status in vivo. Therefore, many studies have been performed evaluating TAC as a synergistic reaction. A depletion of total antioxidant capacity was observed in coronary artery disease patients [93,99]. Some contrasting results were reported, showing higher levels of serum TAC in patients with atherosclerosis [100]. The contrast is apparent, since a key component of TAC is uric acid, which has been demonstrated to be associated with cardiovascular disease [101,102,103]. Moreover, as it will be discussed below, the interpretation of TAC could also be related to the natural history of the diseases, since an initial increase, in response to OS, is followed by a depletion and therefore a reduced plasma value. 

Finally, TAC is influenced by dietary habits. While a high energy diet could determine an increase in ROS, especially at the mitochondrial level [86,104], a beneficial effect could be exerted by natural antioxidants contained in fruit and vegetables [105]. It has been reported that the plasma levels of oxidized LDL negatively correlate lutein, β-carotene, and TAC in relation to fruit/vegetable intake [106]. The levels of malondialdehyde (MDA) was higher in the group of obese participants with lower fruit and vegetable intake [107]. Some studies look into on the effects of dietary intervention based on augmented antioxidant contribution [105], but they are focused on the amelioration of targets of OS rather than the evaluation of endogenous TAC. 

As reported above, MetS and adult growth hormone deficiency (GHD) share different biochemical features, such as insulin resistance (IR) but differ in the antioxidant pattern [108]. On the other hand, immunoglobulin free light chains (FLCs) are molecules with biological functions that are still relatively unknown, but they represent a marker of chronic inflammation. Insulin resistance, however, may reveal different scenarios that cannot overlap entirely. Thus, we compared different phenotypes of IR: patients with GHD, patients with MetS, and patients with hyperferritinaemia. The parameters of inflammation and oxidative stress were significantly different in GHD group vs. the other two groups. These data confirm that different mechanisms of oxidative stress and inflammation underlie these three subtypes of IR and could be used to classify different phenotypes of this complex syndrome [108]. Other experiments extending this observation are reported in Supplementary Materials (see Figure S1). According to this last experiment, we also investigated a larger group of hyperferritinaemia (n = 42) including subjects (n = 16) with genetic alterations for hemochromatosis (mutations of C282Y, H63D, S65D). This study confirmed that LAG is inversely correlated with plasma ferritin concentrations (ferritinemia), suggesting a higher consumption of antioxidants in more severe forms of hemochromatosis (Figure S1).

## 5. High TAC as Response to Augmented Radical Formation

Adult growth hormone deficiency (GHD) is being increasingly recognized as a cause of premature death, due to metabolic, cardiovascular, and oncologic causes, exacerbated by oxidative stress [109,110,111,112,113,114,115,116]. In our personal clinical experience, the diagnosis of GHD is under evaluated, due to its nonspecific clinical picture and the strict guidelines that limit the possibility on testing this condition. As reported above, it partially overlaps with MetS, but with specific features [112]; despite this, the topic of OS is poorly investigated. In GHD condition, oxidative stress, together with low-grade inflammation, dyslipidemia, pro-thrombotic state, and impaired adipokine profile, could contribute to augmented cardiovascular risk [117,118,119,120]. The GH-IGF1 axis influences the production and release of NO at the endothelial level, protecting it from atherogenic processes, but beneficial effects on the myocardial structure and metabolism have also been demonstrated [121,122,123,124,125]. Therefore, it is not surprising that ventricular dysfunction and heart failure are associated with GHD [118,119].

As far as oxidative stress is concerned, an early study demonstrated increased levels of lipid-derived free radicals in GHD, with an improvement in this parameter observed after replacement therapy [120]; the role of OS, on the contrary, was not confirmed in another study [121]. Different studies have searched for the biomarkers of inflammation in GHD, including increased leptin [122], low adiponectin [123], increased adipsin [111], and increased lipocalin [124]. However, some of these observations were considered as adaptive or a simple consequence of increased adipose tissue [118]. Despite this, both in normal-weight and obese GHD patients, a marked increase in C reactive protein has been described [125]; also, pro-inflammatory cytokines may increase cardiovascular risk, mainly acting at the endothelial level [126,127]. In experimental models, such as diabetic cardiomyopathy, the overproduction of ROS and, consequently, cellular calcium overload are related to the development and progression of the disease [128]; a role has been attributed to the low expression of Nrf2 in such animals and also the downregulation of antioxidant genes [129].

We conducted a case–control observational study with the primary objective of evaluating new parameters of oxidative stress and macromolecular damage in adult subjects with growth hormone deficiency (GHD). Additionally, we included subjects with partial GHD, a syndrome with undefined boundaries and unknown impact on cardiovascular risk, along with control subjects [130]. We assessed serum nitro-tryptophan and urine di-tyrosine as parameters of protein damage, urinary hexanoyl-lysine as an index of lipid damage, and urinary 8-OH-deoxyguanosine as a parameter of DNA damage; a correlation with TAC was also investigated. The various parameters showed diversified patterns: values of 8-OH-deoxyguanosine did not significantly differ among the three groups; similarly, no significant difference was found in the nitro-tryptophan serum levels. Interestingly, the values of hexanoil-lysine exhibited a tendency to increase when comparing total vs. partial GHD and controls, although it was not significant. On the contrary, significantly lower levels of di-tyrosine in partial vs. total GHD and controls were found. Additionally, significantly greater values of TAC were observed in total and partial GHD vs. controls. Thus, our results confirm that oxidative stress is increased both in partial and total adult GHD. The apparent amelioration of proteic damage seemed to be related to an increased antioxidant response; however, despite a further increase in LAG in total GHD, this appeared as an inadequate compensation exerted by antioxidants, which may be connected to the complications associated with this rare disorder [12].

In another study, we aimed to evaluate DNA oxidative damage by analyzing the production of thymidine-glycol in lymphocytes and its correlation with TAC in the same three groups (partial and total GHD, and controls). Unexpectedly, cellular thymidine-glycol was lower in the total GHD group, but was accompanied by a significant increase in plasmatic TAC. We hypothesized that in the adult GHD condition, the production of antioxidant species could exert a protective effect on thymidine-glycol formation in response to increased oxidative stress, and consequently on DNA intracellular damage. The open field remains the relationship between plasma and tissue compartments; however, this pilot study could be inserted in the complex scenario of oxidative damage of GHD, a subtle, yet poorly defined condition, worthy of further investigation [131].

## 6. Treatments for Oxidative Distress

### 6.1. Dietary Interventions 

Many different diet regimes with high levels of natural antioxidant have been proposed [132,133]. As discussed above, OS could play a role in metabolic syndrome-related manifestations contributing to insulin resistance (IR). Several trials with supplementation with natural antioxidants, including vitamins C and E, carotenoids, coenzyme Q 10, arginine, alpha lipoic acid and selenium, are reported in the literature [134,135,136,137]. They show an improvement in obesity and the related complications. However, these study do not consider TAC values as a biomarker of OS. 

We previously demonstrated the beneficial effects of a natural antioxidant-enriched diet in patients with metabolic syndrome [138]. A personalized program, with a calculated antioxidant intake of 800–1000 mg/day from fruits and vegetables, was supplemented, resulting in a significant decrease in HOMA and the insulin peak response to glucose load, despite a similar decrease in BMI compared to a hypocaloric diet. Moreover, the insulin response, measured as area under curve, showed the greatest decrease when coupled with metformin. Therefore, we suggested that dietary antioxidants ameliorate insulin-sensitivity in obese subjects with IR by enhancing the effect of insulin-sensitizing drugs albeit the molecular mechanisms are yet unclear [138]. Fresh fruit and vegetables are not the only natural antioxidants in diet regimen. Previous studies have shown that a diet enriched with tree nuts is able to reduce HbA1c in diabetic patients. This positive result is probably related to the antioxidant properties of such a diet [139]. In a preliminary longitudinal study comparing two types of dietary antioxidant treatments, we included three groups of patients with MetS under the following protocols: a standard hypocaloric diet, a hypocaloric diet enriched with fruits and vegetables, and a hypocaloric diet enriched with mixed dried fruits (20 g of almonds and 30 g of tree nuts). All groups exhibited a similar decrease in BMI, but only the antioxidant-enriched diets led to a reduction in insulin levels. Additionally, only the third group showed an increase in HDL cholesterol.

Taken together these preliminary data suggest that there is a differential metabolic response to various regimens of natural antioxidant-enriched diets even if physiopathological mechanisms remain to be further elucidated [140]. 

### 6.2. Bariatric Surgery 

When morbid obesity cannot benefit from a dietary/exercise treatment, bariatric surgery can be recommended. After techniques inducing malabsorption and reduction in absorptive surface, to the detriment of severe side effect, biliopancreatic diversion (BPD) has been practiced for a long period. It consists of partial gastrectomy with Roux-en-Y reconstruction [141]. As a consequence, food in the digestive tract does not undergo the normal action of biliary and pancreatic secretions, and patients develop fat malabsorption and a partial starch malabsorption, while maintaining absorption of mono-disaccharides and proteins [142]. In a previous study, because of lipid malabsorption, we evidenced a marked reduction in lipid antioxidant Coenzyme Q10 [28]. The mechanisms leading to this reduction (weight loss per se, lipid malabsorption, metabolic, or hormonal variations) are not yet clear.

Subsequently, other less-invasive techniques have been introduced, including gastric bypass (GB) and mini-gastric bypass (mGB). We therefore started a prospective longitudinal pilot study to evaluate long term TAC (12–18 months) in the three employed techniques (BPD, GB, mGB) [143]. We present some preliminary data in the Supplementary Materials. Despite BPD having different metabolic parameters and being influenced by the surgery procedure, in comparison to the other techniques, due to the prevalent lipid malabsorption a significant marked decrease in HOMA and increase in HDL-cholesterol were observed in mGB vs. the other two techniques. Moreover, we found a decrease in TAC, expressed as LAG, in all the groups (see Figure S2 in Supplementary Materials). These preliminary results suggest that body weight reduction per se, rather than the different surgical procedures, can be responsible for TAC values amelioration. We can also suggest that these data, which could indicate a reduction in OS and therefore a reduction in compensatory antioxidant response, could appear as a promising index; however, with a broader perspective, it could be a mechanism contributing to the well-known phenomenon of weight regain. Moreover, we propose that the total antioxidant capacity should be determined in patients that are undergoing bariatric surgery, despite this not being a routine measure that is recommended [144] and, in the case of persistent reduction, supplemented according to a personalized approach. Further study can gain insight into the differential patterns of specific antioxidants that can contribute to the antioxidant power in serum of obese subjects. 

## 7. Perspective on Future Clinical Implication and Conclusions

Here, we aimed to shed light on the important role of TAC. In fact, the determination of TAC is an inexpensive and simple method to evaluate the components of antioxidants, such as non-proteic, non-enzymatic small chain-breaking substances in plasma and other biological fluids. However, the interpretation of this parameter should be carefully considered in the specific clinical context and, if possible, together with others parameters of oxidative damage. 

In summary, three clinical implications of the above-reported observations concerning TAC can be pinpointed: (1) the physiopathological deepening of etiology and clinical course of illness connected to OS; (2) the diagnostic role based on the evaluation of oxidative conditions; and 3) the appropriate treatment when endogenous antioxidants are inadequate. Thus, all these data, as a whole, despite their limitations, could represent the basis for treatment with natural or chemical antioxidants as a complement to ordinary pharmacological treatment.

## Data Availability

All data are available in the paper.

## Supplementary Materials

The following supporting information can be downloaded at https://www.mdpi.com/article/10.3390/antiox13080933/s1, Figure S1: Correlation between LAG and ferritinemia. TAC values, expressed as LAG (in seconds), are inversely correlated with plasma ferritin concentrations (ng/mL). The antioxidant capacity afforded by chain-breaking antioxidants is here reported as the length of lag phase (LAG) in seconds; Figure S2: The mean of TAC values, expressed as LAG (in seconds), in the three groups before (Pre) and after underwent bariatric surgery (Post): gastric bypass (GB), biliopancreatic diversion (BPD), mini-gastric bypass (mGB), and all groups (All).
