# Supplementary material for: The Double-Edged Sword of Total Antioxidant Capacity: Clinical Significance and Personal Experience
Source: Antioxidants (Basel). 2024 Aug 1;13(8):933. doi: 10.3390/antiox13080933 (PMC11351343; doi:10.3390/antiox13080933)
Supplement: Supplementary file 1 [file antioxidants-13-00933-s001.zip › antioxidants-3101262-supplementary.pdf]

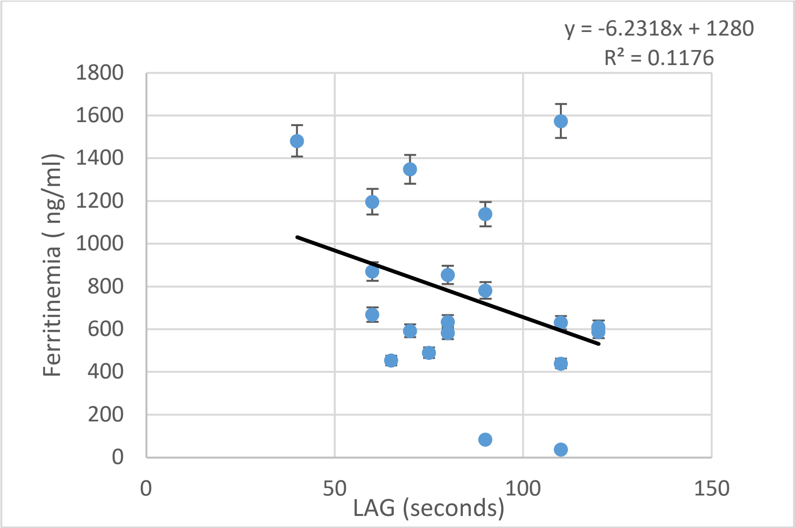


**Figure S1.** Correlation between LAG and ferritinemia. TAC values, expressed as LAG (in seconds), are inversely correlated with plasma ferritin concentrations (ng/ml). The antioxidant capacity afforded by chain-breaking antioxidants is here reported as the length of lag phase (LAG) in seconds.

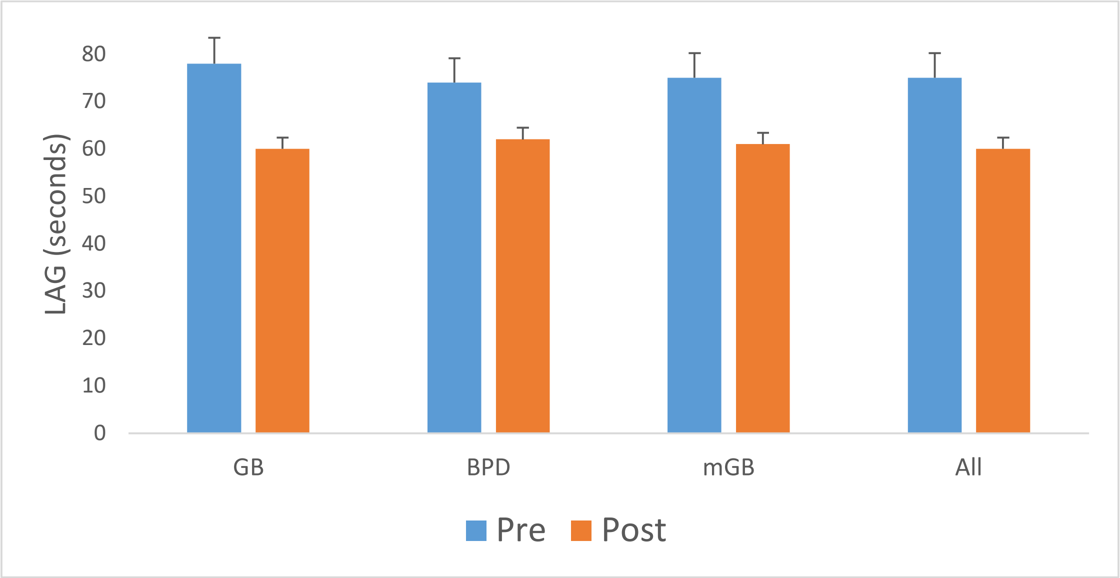


**Figure S2.** The mean of TAC values, expressed as LAG (in seconds), in the three groups before (Pre) and after underwent bariatric surgery (Post): gastric bypass (GB), biliopancreatic diversion (BPD), mini-gastric bypass (mGB), and all groups (All).
